# Supplementary material for: Coherent manipulation of valley states at multiple charge configurations of a silicon quantum dot device
Source: Nat Commun. 2017 Jul 5;8:64. doi: 10.1038/s41467-017-00073-x (PMC5498670; doi:10.1038/s41467-017-00073-x)
Supplement: Supplementary file 1 — Supplementary Information [file 41467_2017_73_MOESM1_ESM.pdf]

File name: Supplementary Information
Description: Supplementary Figures, Supplementary Notes and Supplementary References

File name: Peer Review File
Description:

Supplementary Note 1 Qubit Excitation at Multiple Charge Configurations

We can perform qubit operation in multiple charge configurations of the QD. For example, similar coherent oscillation patterns have been observed for two consecutive anti-crossings, corresponding to the first two observed electrons in the left QD. The analysis of the other anti-crossing's qubit behavior proceeds just as that of the anti-crossing which was examined in the main text. Supplementary Fig. 1 shows qubit operation in the (1,0,1) and (2,0,1) regions. There are significant qualitative similarities between the two tuning points. The two pulse height versus pulse width scans can be compared in Supplementary Figures 1b and 1c. The alternative crossing's decoherence dependence can be seen in Supplementary Fig. 1d and the different dispersion can be seen in Supplementary Fig. 1e. The energy diagram for the (1,1) to (2,0) transition is more complex since it involves two electrons.[1] Supplementary Figure 1f shows such a diagram for the lowest energy states. For operation as a valley qubit, the spin degree of freedom is not important since the qubit operation involves only the low-lying energy states, $S_{v1v1}(2,0)$ and $T_{v1v2}(2,0)$, which has a gap of δ, similar to that between $|L_{v_1}\rangle$ and $|L_{v_2}\rangle$ in the (0,1)-(1,0) case.

We would like the note here that the broad, bright line in Supplementary Fig. 1b is due to an incoherent process,[2] which can be observed with and without the coherent oscillations. When pulsed into the reconfiguration line, the two levels of the QDs are detuned, so the electron can then tunnel between the two QDs. Due to the large inter-dot tunneling rate, the probabilities for the electron to be in the two QDs are equal. In fact, we found that the strength of this line depends on the inter-dot tunneling rate.

Supplementary Note 2 Effect of Tunneling to 2DEG

Pulse-induced tunneling during the operation time and the effect on apparent coherence time.

One difference between our hybrid qubit and that of some other groups is that we do not exhibit the detuning independent coherence times at greater pulse heights, which should be a natural consequence of the rather flat dispersion regime that the hybrid qubit operates in. We believe that this is due to our pulsing scheme, which is usually limited to a single gate, rather than being composed of two coordinated pulses on different gates, as seen in Supplementary Fig. 2c. Because of this, as the amplitude of the pulse increases, the tendency to tunnel from (1,0) to (1,1) during the operation time becomes greater. If this happens, it disrupts the operation and initialization and the signal appears to more quickly attenuate as the pulse gets deeper into the region where (1,1) is favored. This can be observed in Supplementary Fig. 2d. When two counteracting pulses are placed on separate gates, as in Supplementary Fig. 2a, the general strength of the signal is greater and the coherent region extends further in ε as seen in Supplementary Fig. 2b. Due to difficulties with syncing the two pulses, the bulk of the experiments are performed with single-gate pulses. But the increased coherence time associated with two-gate pulses suggests that the discrepancy between the detuning dependence of our coherence times and those of other groups is due to our pulsing limitations, and were we better able to sync the pulses, a similar coherence profile may be observed.

An alternative possibility is that the greater pulses begin to have an effect on the barrier potential and not just the plunging voltage. By lowering the barrier, the electrons in the left dot are exposed to the environment to a greater extent, which contributes to dephasing during operation.

The high visibility region

The other major effect of tunneling to the 2DEG is to enhance the sensitivity of the QPC to pulse induced effects by trapping the system in the (0,0) state if the electron is measured to have remained in (1,0) at the end of the pulse. For this to happen, the measurement point, which is in the (0,1) region of the stability diagram, must correspond to a configuration where the (0,0) state is energetically preferable to (1,0). When viewed in the stability diagram, this region corresponds to the part of (1,0) within the triangular region bounded by the (0,0)-(0,1) line and the extension of the (0,0)-(1,0) line.[3] (All three regions in Supplementary Fig. 3a) Furthermore, the tunneling from the (1,0) state to the (0,0) must usually occur faster than the natural charge relaxation time from (1,0) to (0,1). In our experiments, the typical charge relaxation time is about 10-15 ns. Also, the tunneling from the (0,0) state to the (0,1) state must occur slowly enough to have a measurable effect on the QPC, but fast enough that it does not disrupt initialization back into the (0,1) state prior to the beginning of the next pulse. The region where all of these conditions are met is the part of the stability diagram where interference fringes can be observed.

Supplementary Fig. 3 shows different re-initialization pathways at different working points of the stability diagram. In the work presented here, visibility is limited to a trapezoidal region made up of only regions 1 and 2 of Supplementary Fig. 3a. In region 1, the mechanism of relaxation is the same as for the standard charge qubit high visibility region: the electron in the excited charge state, (1,0), tunnels out of the dot and another tunnels back in to fill the ground state (Relaxation pathways outlined in Supplementary Fig. 3b). However, when the measurement point is further detuned towards the middle dot, the electron measured to be in the excited charge state makes an adiabatic transition to an excited state of the middle dot, as indicated by the dotted arrow in Supplementary Fig. 3c. From here relaxation is only possible if it is energetically favorable for the electron to tunnel out of the middle dot, as it is in region 2 but not region 3. The total absence of fringes in region 3, where tunneling assisted relaxation is not energetically feasible, indicates a relaxation time between the two middle dot states that is significantly longer than the pulse repetition rate. This is consistent with prior literature[6-8] suggesting that valley to valley relaxation time is on the order of tens to hundreds of microseconds. The experiments presented in this paper were primarily performed in region 1.

Supplementary Note 3 Hamiltonian parameter extraction

Simulation of Lock-In Signal

A lock-in current signal will be given by

$$\mathrm{Re}[I_{\mathrm{lock-in}}] \quad = \int I\left(\theta\big(V + A\sin(\phi)\big)\right)\sin(\phi)\,\mathrm{d}\phi \quad (1)$$

where the measured current, I, is a function of parameters θ, which in turn depend on the fixed side gate bias voltage, V. The quantity A is the lock-in voltage excitation amplitude.

In the case of our setup, I is the average QPC signal over many pulse trains. The parameters of import, θ, are ε_{p}, and t_{p}. Since $\varepsilon_{\mathrm{p}} = \varepsilon_0 + \varepsilon_{\mathrm{nom}}$, and $\varepsilon_{\mathrm{nom}}$ is determined by the pulse generator and not the fixed voltage of the side gates, ε_0 is the only parameter that is affected by the excited voltage. So, we may write, $\varepsilon_0(\phi) = \hat{\varepsilon}_0 + \alpha A \sin(\phi)$. Therefore

$$\begin{aligned} &\mathrm{Re}\left[I_{\mathrm{lock-in}}\left(t_{\mathrm{p}}, \varepsilon_{\mathrm{nom}}, \hat{\varepsilon}_0\right)\right] \\ = \quad &\int I\left(t_{\mathrm{p}}, \varepsilon_{\mathrm{nom}}, \hat{\varepsilon}_0 + \alpha A \sin(\phi)\right) \sin(\phi)\, \mathrm{d}\phi \\ \approx \quad &\sum_{\phi_i = -\frac{\pi}{2}}^{\frac{\pi}{2}} I\left(t_{\mathrm{p}}, \varepsilon_{\mathrm{nom}}, \hat{\varepsilon}_0 + \alpha A \sin(\phi_i)\right) \sin(\phi_i) \quad (2) \end{aligned}$$

Where, each of the terms $I\left(t_{\mathrm{p}}, \varepsilon_{\mathrm{nom}}, \hat{\varepsilon}_0 + \alpha A \sin(\phi_i)\right)$ may be computed in a separate simulation. This became the simulated lock-in signal.

Contrast with other reported hybrid systems

According to the fit for the parameters of the Hamiltonian, with $\alpha = 3\%$: $\delta = 5.57$ GHz , $\Delta_1 =$ 6.4 GHz and $\Delta_2 = 13.6$ GHz. In comparison to other hybrid like systems, ours operates in a different regime of tunnel coupling. Those other systems tended to operate where $2\Delta_1$ is less than δ. For example, Shi et al.4 reports values where $\Delta_1 = 2.62$ GHz, $\Delta_2 = 3.5$ GHz, and $\delta = 9.2$GHz (See also Kim et al.[5]). The spectrum which results from such a regime is different from that reported here. Most notably, the avoided crossing between $|\mathrm{M}\rangle$ and $|\mathrm{L}_{\mathrm{v}_1}\rangle$ at $\varepsilon \approx 0$ has a small enough splitting that passage through it is non-adiabatic. This means that coherent oscillations can be seen even when ε_{p} is close to 0. The oscillations seen at $\varepsilon_{\mathrm{p}} \approx 0$ are slower than those caused by δ and are protected from decoherence because $\frac{\mathrm{d}\Delta E_{12}}{\mathrm{d}\varepsilon} = 0$ at that point. In contrast our system has no visible fringes as ε_{p} approaches 0. We conclude that this is because when $0 < \varepsilon_{\mathrm{max}} < \varepsilon_{\mathrm{x}}$, the operation includes no non-adiabatic process. Furthermore, at that point and anywhere to the left of ε_{x}, $\frac{\mathrm{d}\Delta E_{12}}{\mathrm{d}\varepsilon}$ is large, contributing to significant susceptibility to electronic noise. The conclusion that we operate in a regime where $2\Delta_1, 2\Delta_2 > \delta$, is further supported by the results of numerical fitting for the Hamiltonian parameters.

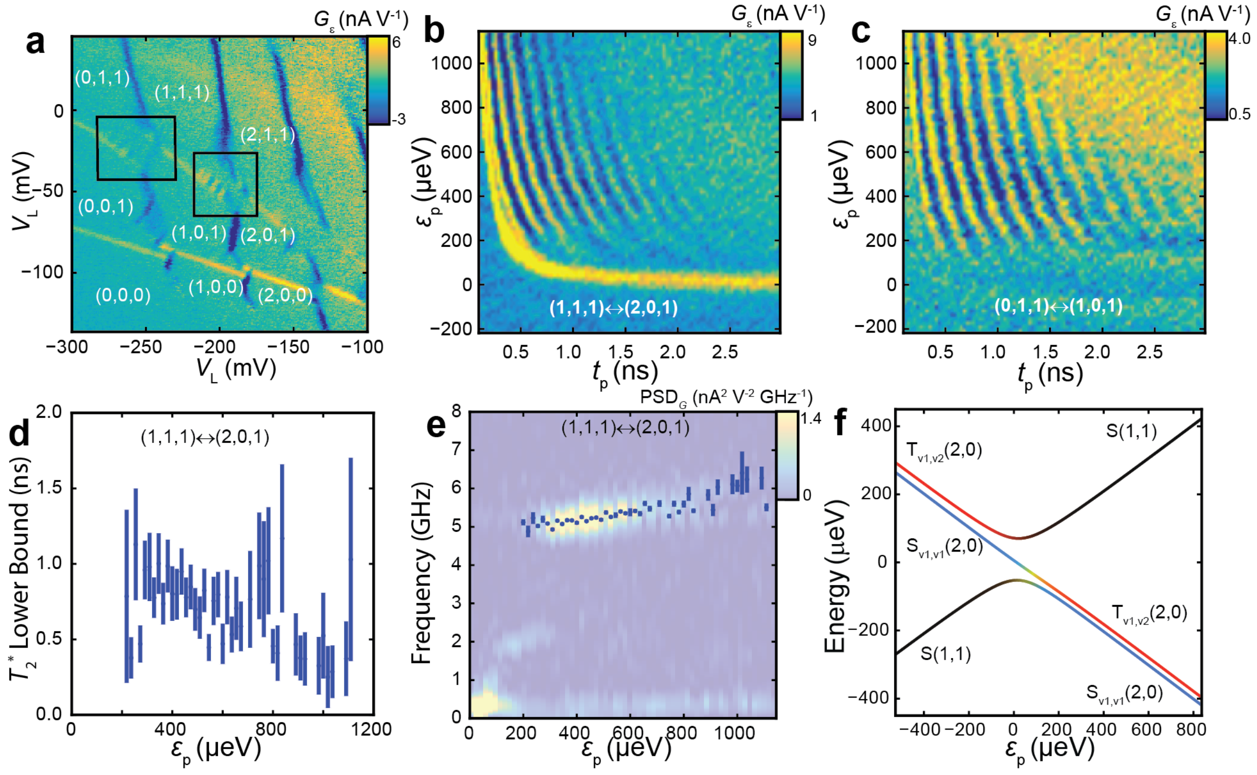

Supplementary Figure 1: Details of Second Charge Configuration a. The triple dot charge stability diagram with a pulse applied: This figure covers the same range as the stability diagram in Main Text Fig. 1b, but with a pulse applied to V_{L}. It can be seen that interference fringes are simultaneously present at both the $(0,1,1) \leftrightarrow (1,0,1)$ transition and the $(1,1,1) \leftrightarrow (2,0,1)$ transition. **b.** Experimental mapping of the detuning dependence of the time domain oscillations at the $(1,1,1) \leftrightarrow (2,0,1)$ transition. This is directly analogous to Main Text Fig. 3a. **c.** The pulse width and height dependence of the $(0,1,1) \leftrightarrow (1,0,1)$ transition: This is the same as Main Text Fig. 3a. It is repeated here for comparison with (b). **d.** The extracted decay times and **e.** frequencies: These are obtained from Supplementary Fig. 1a in the same way as the plots in Main Text Fig. 4. **f.** The fitted spectrum for the $(1,1,1) \leftrightarrow (2,0,1)$ transition where the three relevant states are identified with the lowest lying two electron states.

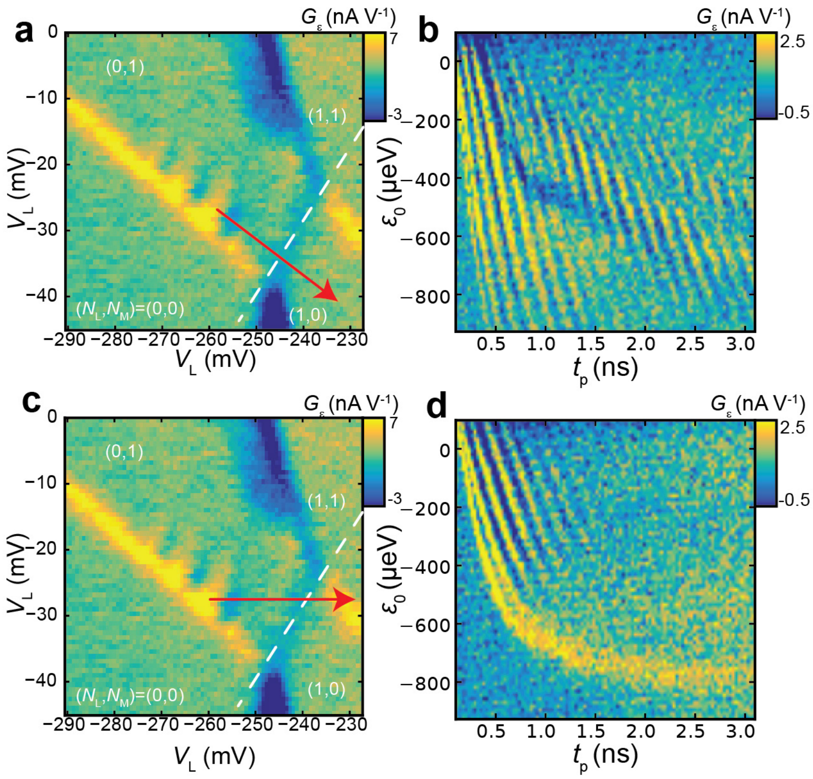

Supplementary Figure 2: Effect of Double Gate Pulsing a. Stability diagram from Main Text Fig. 1a showing the direction of a pulse when voltages are applied to both V_L and V_R: The effect is to have a pulse which is more purely in the detuning direction without bringing the system to a voltage space where the ground state has a different total occupation. **b.** Demonstration of enhanced coherence times with a diagonal pulse: The initialization/measurement point is varied as pulse width traces are taken with a fixed pulse height of +30 mV on V_L and -30 mV on V_R. When such a pulsing system is used, strong oscillations are clearly visible out to 3 ns, which is considerably longer than the longest achieved with a single gate pulse. However, due to difficulty in pulse synchronization, usually only a single gate pulse is used. **c.** A stability diagram showing the direction of a single gate pulse: Such a pulse brings the system into the (1,1) region during operation. **d.** A plot identical to that of (b) except that pulses are only applied to V_L: A reduced apparent coherence time is visible as well as an additional bright and apparently incoherent line, which is believed to be $(1,1) \leftrightarrow (1,0)$ charging line.

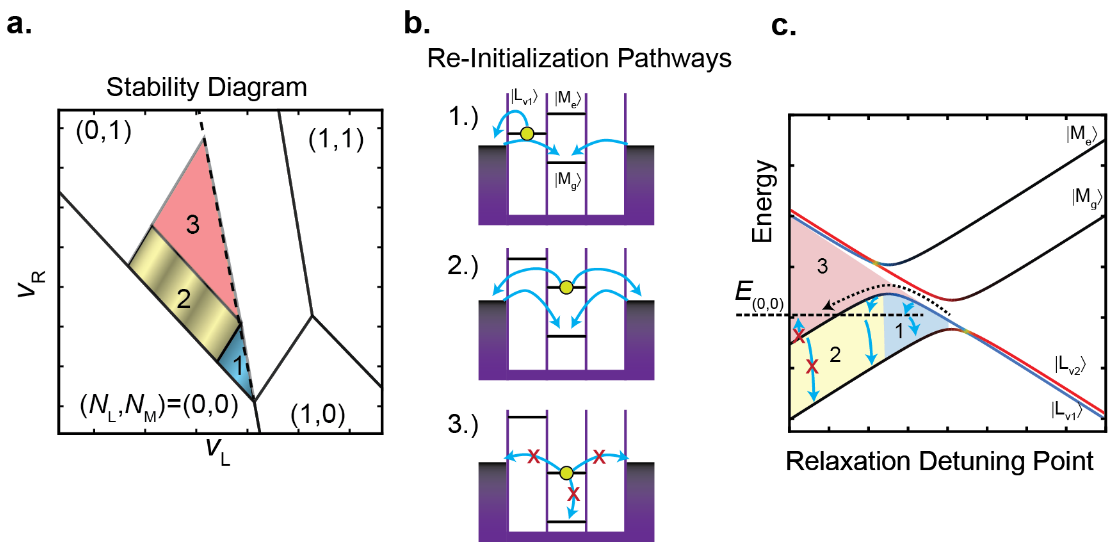

Supplementary Figure 3: Trapezoidal Visibility Region a. The standard high visibility region is divided into three regions. Fringes are only observed in regions 1 and 2. **b.** The relaxation pathways of the three regions: In region 1, an electron measured to be in the excited state is in a different charge configuration. The electron tunnels out and then a different electron tunnels back in. Also, direct charge relaxation is possible on the timescale of the pulse repetition rate. In regions 2 and 3, the electron will be in an excited middle dot state. In region 2, tunneling out before resetting is energetically favorable, allowing for visible fringes. In region 3 such tunneling is not allowed. Furthermore, valley to valley relaxation is slow compared to the pulse repetition rate. Therefore, resetting is disrupted and no fringes are visible. **c.** A spectrum showing the excited middle dot state as well as the state trajectory when the system does not return to the middle ground state (dotted arrow). The shape of the spectrum is reflected in the trapezoidal region of visibility. The regions are defined on the x axis by the relaxation detuning point and on the y axis by the relative energy of the (0,0) state, $E_{0,0}$.

Supplementary References

1. Maune, B. M. et al. Coherent singlet-triplet oscillations in a silicon-based double quantum dot. *Nature* **481**, 344–347 (2012).

2. Harbusch, D., Manus S., Tranitz, H. P., Wegscheider, W., and Ludwig, S., Radio frequency pulsed-gate charge spectroscopy on coupled quantum dots, *Phys. Rev. B* **82**, 195310 (2010).

3. Petersson, K. D., Petta, J. R., Lu, H. & Gossard, A. C. Quantum coherence in a one-electron semiconductor charge qubit. *Phys. Rev. Lett.* **105**, 246804 (2010).

4. Shi, Z. et al. Fast coherent manipulation of three-electron states in a double quantum dot. *Nat. Commun.* **5**, 3020 (2014).

5. Kim, D. et al. Quantum control and process tomography of a semiconductor quantum dot hybrid qubit. *Nature* **511**, 70–74 (2014).

6. Wang, K., Payette, C., Dovzhenko, Y., Deelman, P. W. & Petta, J. R. Charge Relaxation in a Single-Electron Si/SiGe Double Quantum Dot. *Phys. Rev. Lett.* **111**, 046801 (2013).

7. Kawakami, E. et al. Electrical control of a long-lived spin qubit in a Si/SiGe quantum dot. *Nat. Nanotechnol.* **9**, 666-670 (2014).

8. Kawakami, E. et al. Gate fidelity and coherence of an electron spin in an Si/SiGe quantum dot with micromagnet. *Proc. Natl. Acad. Sci.* **113**, 11738-11743 (2016).
